# HIV-Related Arterial Stiffness in Malawian Adults Is Associated With the Proportion of PD-1–Expressing CD8^+^ T Cells and Reverses With Antiretroviral Therapy

**DOI:** 10.1093/infdis/jiz015

**Published:** 2019-01-09

**Authors:** Christine Kelly, Henry C Mwandumba, Robert S Heyderman, Kondwani Jambo, Raphael Kamng’ona, Mishek Chammudzi, Irene Sheha, Ingrid Peterson, Alicja Rapala, Jane Mallewa, A Sarah Walker, Nigel Klein, Saye Khoo

**Affiliations:** 1 Insitute of Translational Medicine, University of Liverpool; 2 Department of Clinical Sciences, Liverpool School of Tropical Medicine, Liverpool; 3 Division of Infection and Immunity; 4 Institute for Cardiovascular Sciences; 5 MRC Clinical Trials Unit; 6 Institute of Child Health, University College London, London, United Kingdom; 7 Malawi–Liverpool–Wellcome Trust Clinical Research Programme, College of Medicine; 8 Department of Medicine, Queen Elizabeth Central Hospital, Blantyre, Malawi; 9 HIV Molecular Research Group, University College Dublin, Ireland

**Keywords:** HIV, PD-1, T-cell exhaustion, sub-Saharan Africa, endothelial damage, arterial stiffness, cardiovascular disease

## Abstract

**Background:**

The contribution of immune activation to arterial stiffness and its reversibility in human immunodeficiency virus (HIV)–infected adults in sub-Saharan Africa is unknown.

**Methods:**

HIV-uninfected and HIV-infected Malawian adults initiating antiretroviral therapy (ART) with a CD4^+^ T-cell count of <100 cells/μL were enrolled and followed for 44 weeks; enrollment of infected adults occurred 2 weeks after ART initiation. We evaluated the relationship between carotid femoral pulse wave velocity (cfPWV) and T-cell activation (defined as HLA-DR^+^CD38^+^ T cells), exhaustion (define as PD-1^+^ T cells), and senescence (defined as CD57^+^ T cells) and monocyte subsets, using normal regression.

**Results:**

In 279 HIV-infected and 110 HIV-uninfected adults, 142 (37%) had hypertension. HIV was independently associated with a 12% higher cfPWV (*P* = .02) at baseline and a 14% higher cfPWV at week 10 (*P* = .02), but the increases resolved by week 22. CD4^+^ and CD8^+^ T-cell exhaustion were independently associated with a higher cfPWV at baseline (*P* = .02). At 44 weeks, arterial stiffness improved more in those with greater decreases in the percentage of CD8^+^ T cells and the percentage of PD-1^+^CD8^+^ T cells (*P* = .01 and *P* = .03, respectively). When considering HIV-infected participants alone, the adjusted arterial stiffness at week 44 tended to be lower in those with higher baseline percentage of PD-1^+^CD8^+^ T cells (*P* = .054).

**Conclusions:**

PD-1^+^CD8^+^ T-cells are associated with HIV-related arterial stiffness, which remains elevated during the first 3 months of ART. Resources to prevent cardiovascular disease in sub-Saharan Africa should focus on blood pressure reduction and individuals with a low CD4^+^ T-cell count during early ART.

## Teaser

Arterial stiffness is increased in Malawian adults with low CD4 during the first 3 months of antiretroviral therapy, compared to adults without HIV. Hypertension is an important traditional risk factor and immune activation, including CD8 exhaustion, also contributes to arterial stiffness.

Sub-Saharan Africa (SSA) has the highest burden of human immunodeficiency virus (HIV) infection worldwide, with 25.6 million people living with the disease [1]. The region also faces an accelerated epidemic of noncommunicable diseases, including cardiovascular disease (CVD) [2]. Mortality from CVD is high in SSA, causing an estimated 1 million deaths/year [3]. This rate is predicted to increase, with noncommunicable disease–related mortality estimated to surpass infection-related deaths by 2030 [3]. Studies from high-income settings suggest the risk of CVD is approximately doubled in people living with HIV, even after adjustment for confounders such as socioeconomic status, traditional cardiovascular risk factors, and viral hepatitis virus coinfection [4, 5]. Several processes may contribute, including a high HIV load; side effects of antiretroviral therapy (ART), particularly protease inhibitor treatment; and the effects of chronic immune activation despite effective ART [6]. Immune activation may be driven by persistent low-level HIV viremia, microbial translocation, and subclinical infections [7, 8]. However, the risk of CVD in people living with HIV in SSA has not been well characterized. It is likely that immune activation differs in low-income settings, owing to the effects of more-advanced HIV disease at presentation, more-frequent acute coinfections, and malnutrition with disruption to the gut barrier [9, 10]. Traditional cardiovascular risk factors also vary, with hypertension being more prevalent than diabetes, dyslipidemia, or obesity, although with epidemiological transition and increasing urbanization in the region, this may change in the near future [11, 12].

Large cohorts documenting cardiovascular events have not been established in SSA, limiting the assessment of cardiovascular risk. One physiological marker of cardiovascular risk is the carotid femoral pulse wave velocity (cfPWV), a gold standard measurement of arterial stiffness [13–15]. Although adjusted for concurrent blood pressure, the reading can be affected by blood viscosity and ambient temperature [13]. Nevertheless it has been shown to be reliable and reproducible and has been validated against clinical outcomes in high-income settings [16–18]; a cfPWV in the top versus bottom tertile is associated with a >2-fold increased risk of myocardial infarction/stroke [18]. The 2007 European Society of Cardiology consensus guidelines proposed a 12-m/second threshold as high risk for CVD events [14]. A few small studies have assessed arterial stiffness in people living with HIV in low-income areas of SSA [19–23], but none have evaluated the impact of chronic immune activation over time. This study therefore aimed to characterize the contribution of immune activation to arterial stiffness in HIV-infected Malawian adults initiating ART with advanced immunosuppression, compared with that in HIV-uninfected adults, and to determine how this changed over time on ART.

## METHODS

### Study Design

Adults aged >18 years presenting for HIV testing at the voluntary testing clinic, the HIV outpatient clinic, and the medical inpatient wards at the Queen Elizabeth Central Hospital (Blantyre, Malawi) were recruited into a prospective cohort from January 2014 until June 2015. Adults with a new HIV diagnosis were approached consecutively and were eligible if they were ART naive, had a CD4^+^ T-cell count of <100 cells/μL, and provided informed written consent. Adults who were confirmed to be uninfected with HIV after self-presenting for an asymptomatic HIV test at the same voluntary testing clinic were eligible if they had no current illness; no history of infection in the previous month, based on clinician assessment and medical notes review; and provided informed written consent. Exclusion criteria were living outside the Blantyre area, inability to attend follow-up visits, pregnancy, or being too unwell to participate, as judged by the study clinicians. Because HIV-uninfected participants were younger, an exclusion criterion of <35 years of age was used for this group from March 2014 onward. From January 2014 until January 2015, HIV-infected participants were corecruited with the REALITY trial (clinical trials registration NCT01825031). REALITY assessed interventions to reduce early mortality following ART initiation in those with a CD4^+^ T-cell count of <100 cells/µL. Participants were simultaneously randomly assigned to one of 3 study interventions, in addition to standard triple-drug ART and cotrimoxazole, and also received a 12-week supply adjunctive raltegravir, a package of opportunistic infection prophylaxis, and/or ready-to-use supplementary food [24–26].

At enrollment and 44 weeks later, participants underwent a detailed clinical assessment, including evaluation for traditional cardiovascular risk factors and infection history and collection of fasting blood specimens, with some specimens collected in tubes containing sodium citrate for immunophenotyping. To reduce the burden of study participation and to ensure that starting ART was prioritized in the severely immunosuppressed HIV-infected population, the baseline study visit was conducted at week 2 following ART initiation. cfPWV was assessed for all participants at enrollment and 10, 22, and 44 weeks later. All participants provided informed written consent, and ethical approval was granted by the College of Medicine Research and Ethics Committee, University of Malawi (P.09/13/1464), and by the University of Liverpool Research and Ethics Committee (UoL000996).

### Outcome Measurement

cfPWV was measured using a Vicorder device (Skidmore Medical, London, UK). The distance was the length from the sternal notch to the umbilicus and then the top mid-point of the femoral cuff, multiplied by 0.8 as per consensus guidelines. Wave forms were saved, and a random sample was reviewed by an experienced independent assessor, blinded to HIV status, at 3 time points during the study, to ensure consistent quality. The intraoperator concordance correlation coefficient for 10 participants was 0.99 (95% confidence interval [CI], .96–1.00).

### Immunophenotyping of Peripheral Blood Mononuclear Cells (PBMCs)

For flow cytometry, whole-blood specimens were processed within 4 hours of collection to isolate PBMCs, using Lymphoprep (Axis-Shields-Diagnostics) as previously described [27]. Cells were analyzed using a CyAn ADP 9 color flow cytometer (Beckman Coulter). The T-cell panel included CD3 BV510, CD4 V450, CD38 PE Cy7, HLA-DR AF700, PD-1 APC, and CD57 FITC (all from BD Biosciences) and CD8 PE (Biolegend). The monocyte panel included HLA-DR AF700, CD14 PE Cy7, and CD16 PE (all from BD Biosciences). Anti-mouse Igk isotype control and negative control particles (BD Biosciences) were used for compensation. A standardized gating strategy was followed for T cells (Supplementary Figure 1*A*) and monocytes (Supplementary Figure 1*B*). Monocyte subsets were identified as previously described [28].

### Statistical Analysis

As data validating a clinically relevant cfPWV threshold in SSA were not available, the 12-m/second threshold in European consensus guidance was used to guide sample size calculations [14]. Recruiting 300 HIV-infected and 100 HIV-uninfected participants provided 80% power to detect an odds ratio (OR) of 1.5 associated with HIV, assuming that 25% of HIV-uninfected participants had cfPWV >12 m/second [14]. After enrollment, it was clear that this threshold was rarely reached, and therefore the planned analysis considered the primary outcome, cfPWV, as a continuous variable.

Factors affecting HIV and arterial stiffness were identified a priori, using a causal diagram (Supplementary Figure 2), as either potential mediators (ie, on the mechanistic pathway between them) or confounders (ie, associated with HIV and arterial stiffness but not on the mechanistic pathway). Categorical and continuous variables were compared between HIV-infected and uninfected adults by using χ^2^ and rank-sum tests, respectively. Correlations between continuous variables were compared using Spearman rho. To avoid undue influence from outliers, continuous variables were truncated at their 97.5th and 2.5th percentiles for regression models. Normal linear regression models were constructed, with log_10_ cfPWV (approximately normal) as the outcome and HIV infection as the primary exposure. Confounders and mediators with a univariate *P* value of <.2 for association with cfPWV were considered, and backward elimination (exit *P* = .2) was used to identify a final model. Where 2 variables were strongly collinear, the variable with the strongest univariate association with cfPWV was considered for inclusion in the multivariate model. Independent effects of immunophenotyping parameters on this model were then also assessed. Overall changes in log_10_ cfPWV over the first 44 weeks from enrollment were estimated using random effects models, considering the impact of HIV on cfPWV both at baseline (intercept) and over time (interaction with time). Regression models for cfPWV 44 weeks after enrollment in HIV-infected participants adjusted for baseline cfPWV and factors identified as confounders or mediators at baseline, and they additionally considered the impact of immune parameters. Adjustment for baseline means that these models identify predictors of change from baseline.

All analysis was undertaken using Stata v13.1 (Statacorp, College Station, TX).

## RESULTS

**Figure 1.**
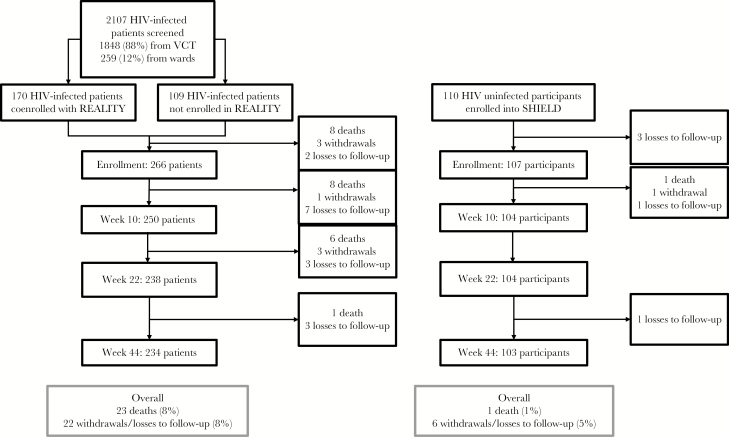
Recruitment flow. Enrollment was performed 2 weeks after screening. HIV, human immunodeficiency virus; VCT, voluntary counseling and testing.

A total of 2107 adults with a new HIV diagnosis were screened, of whom 279 (13%) were recruited. One hundred seventy (61%) were corecruited with the REALITY trial, and 109 (39%) were not (Figure 1). Most exclusions (involving 1477 adults [70%]) were because of a CD4^+^ T-cell count of >100 cells/mm^3^ (complete list of exclusions is in Supplementary Table 1). One hundred ten HIV-uninfected adults were also recruited. Although the HIV-infected and uninfected groups were of similar age (median, 36.6 versus 34.8 years; Table 1), HIV-infected participants were more likely to be male and to have a lower level of education. Three HIV-uninfected adults (3%) versus 5 HIV-infected adults (2%) had a diagnosis of hypertension at enrollment; a further 46 (42%) and 88 (32%), respectively, were discovered to have hypertension during the study. HIV-infected participants had advanced immunosuppression (median CD4^+^ T-cell count, 41 cells/μL; median HIV load, 5.06 log_10_ copies/mL), although only 54 (19%) had World Health Organization (WHO) HIV disease stage 3 or 4.

**Table 1.** Baseline Characteristics, According to Human Immunodeficiency Virus (HIV) Status

Characteristic	Complete Cases	HIV Uninfected (n = 110)	HIV Infected (n = 279)	*P*
Demographic variable				
Age	388	34.8 (30.8–41.2)	36.6 (31.1–43.3)	.41
Female sex	389	66 (60)	122 (44)	.004
Primary school education or less	352	38 (40)	136 (53)	.024
Traditional cardiovascular risk factor				
Weight, kg	375	60 (53–68)	53 (48–59)	<.0001
Waist to height ratio^a^	349	0.47 (0.44–0.53)	0.45 (0.43–0.49)	.0003
BMI^b^	373	21.7 (20.2–25.2)	19.8 (18.3–21.9)	<.0001
Blood pressure, mm Hg				
Systolic	361	128 (114–134)	120 (108–128)	.0001
Diastolic	358	75 (68–82)	73 (68–80)	.27
History of smoking^c^	389	16 (15)	56 (20)	.21
History of alcohol use^c^	389	28 (25)	119 (43)	.002
Preexisting CVD diagnosis	370	1 (1)	1 (0.4)	.47
Prescribed CVD medications	370	5 (5)	4 (1.5)	.08
Preexisting diabetes	367	1 (1.0)	1 (0.4)	.65
Preexisting hypertension	366	3 (3.0)	5 (2.0)	.40
New diagnosis of hypertension	358	46 (42)	88 (32)	.055
Fasting cholesterol level, mmol/L	377	4.0 (3.3–4.5)	3.6 (3.0–4.4)	.049
Fasting glucose level, mmol/L	327	4.6 (4.2–5.2)	4.9 (4.4–5.6)	.015
Creatinine level, µmol/L	381	62 (54–71)	65 (54–78)	.13
Infection-related factor				
Heart rate, beats/min	360	72 (68–80)	82 (72–98)	<.0001
Hemoglobin level, g/dL	375	13.8 (12.7–14.7)	11.4 (10.0–13.0)	<.0001
Current infectious disease at enrollment	377	3 (3)^d^	57 (21)	<.0001
Tuberculosis	…	0 (0)	2 (1)	
Cryptococcal meningitis	…	0 (0)	0 (0)	
Pneumonia	…	0 (0)	10 (4)	
Gastroenteritis	…	1 (1)	17 (6)	
Malaria	…	2 (2)	3 (1)	
Immune-related factor				
Lymphocyte count, ×10^9^ cells/L	370	2.1 (1.6–2.6)	1.2 (0.8–1.7)	<.0001
Monocyte count, ×10^9^ cells/L	323	0.30 (0.25–0.50)	0.40 (0.22–0.60)	.053
Absolute CD4^+^ T-cell count, cells/µL	…	…	41 (18–62)	
HIV load, log_10_ copies/mL	…	…	5.06 (4.62–5.47)	
T-cell activation,^e^ % of cells				
CD4^+^ T cells	193	5 (3–9)	22 (11–34)	<.0001
CD8^+^ T cells	290	11 (6–19)	34 (21–49)	<.0001
T-cell senescence,^f^ % of cells				
CD4^+^ T cells	194	7 (4–9)	15 (9–24)	.0001
CD8^+^ T cells	295	40 (27–53)	54 (44–64)	<.0001
Monocyte phenotype				
Classical (CD14^++^CD16^−^)	263	75 (65–81)	76 (66–83)	.79
Intermediate (CD14^++^CD16^+^)	263	9 (7–14)	10 (6–13)	.10
Nonclassical (CD14^+^CD16^+^)	263	13 (10–22)	14 (9–21)	.59

Data are median value (interquartile range) or no. (%) of participants.

Abbreviations: BP, blood pressure; CVD, cardiovascular disease.

^a^Waist to height ratio measures central obesity, a risk factor for metabolic syndrome [47].

^b^Body mass index (BMI) is calculated as the ratio of the weight in kilograms divided by the height in square meters.

^c^Defined as either a past or current history of regular alcohol or smoking.

^d^Based on results of tests returned after enrollment and at which point the participants reported and physicians confirmed the absence of infection.

^e^Defined as CD38^+^HLA-DR^+^ cells.

^f^Defined as PD-1^+^ cells.

All but 1 HIV-infected participant initiated standard first-line therapy with tenofovir-lamivudine-efavirenz (the exception received zidovudine-lamivudine-nevirapine). In total, 28 (7%) participants withdrew or were lost to follow-up, and 24 (6%) died. Thirteen (29%) were lost to follow-up or died within the first 2 weeks; 16 (36%), between 2 and 10 weeks; and 16 (36%), after 10 weeks (Figure 1). One HIV-uninfected participant died from a hypertension-related intracranial bleed. The 23 deaths in HIV-infected participants were due to pulmonary/disseminated tuberculosis (6), cryptococcal meningitis (3), Kaposi sarcoma (3), gastroenteritis (1), and tuberculous meningitis (1). The cause of death was unknown for 9 participants.

### Arterial Stiffness at Enrollment

At enrollment, the median cfPWV was 7.3 m/second (interquartile range [IQR], 6.5–8.2 m/second) in HIV-infected participants versus 7.2 m/second (IQR, 6.2–8.0 m/second) in uninfected participants (*P* = .07). cfPWV was >12 m/second for 5 patients, of whom 4 had HIV infection.

### Immune Activation at Enrollment

As expected, compared with HIV-uninfected participants, HIV-infected adults had higher proportions of activated (CD38^+^HLA-DR^+^) CD4^+^ and CD8^+^ T cells (*P* < .0001 for both comparisons). HIV-infected adults also had a higher proportion of exhausted (PD-1^+^) CD4^+^ and CD8^+^ T cells (*P* < .0001 for both comparisons). In contrast, there were no differences between HIV-infected and uninfected participants in classical (CD14^++^CD16^−^), intermediate (CD14^++^CD16^+^), and nonclassical (CD14^+^CD16^+^) monocytes (*P* = .79, .10, and .59, respectively; Table 1).

### T-Cell PD-1 Expression Is Independently Associated With HIV-Related Arterial Stiffness 2 Weeks After ART Initiation

Factors univariately associated with baseline cfPWV with a *P* value of < .2 are shown in Table 2A; neither WHO stage nor a diagnosis of acute coinfection were associated with baseline cfPWV (*P* = .25 and .23, respectively). Independently, every 10-year increase in age was associated with an 18% increase in cfPWV (95% CI, 14%–23%; *P* < .0001; univariable associations are in Supplementary Figure 3), with no evidence of an effect modification between age and HIV (interaction *P* = .73). Every 10–mm Hg increase in diastolic blood pressure was also associated with a 9% increase in cfPWV (95% CI, 4%–13%; *P* < .0001), and women had a 9% lower cfPWV (95% CI, 2%–16%) than men (*P* = .001).

**Table 2A.** Predictors of Carotid Femoral Pulse Wave Velocity (cfPWV) Among Adult Malawians at Enrollment

Predictor	Evaluable Adults, No.	Univariate Analysis		Multivariate Analysis Including Confounders (n = 353 Complete Cases)		Multivariate Analysis Including Mediators and Confounders (n = 335 Complete Cases)	
		cfPWV,^b^ Fold Change (95% CI)	*P*	cfPWV,^b^ Fold Change (95% CI)	*P*	cfPWV,^b^ Fold Change (95% CI)	*P*
HIV infection	366	1.09 (.44–2.68)	.06	1.07 (.99–1.16)	.08	1.12 (1.02–1.23)	.02
Potential confounder^b^							
Age (per 10-y increase)	366	1.23 (1.18–1.27)	<.0001	1.18 (1.14–1.23)	<.0001	1.18 (1.13–1.23)	<.0001
Female sex (vs male sex)	366	0.87 (.80–.94)	.001	0.91 (.84–.98)	.01	0.94 (.86–1.02)	.15
Diastolic BP (per 10–mm Hg increase)	353	1.13 (1.09–1.18)	<.0001	1.09 (1.04–1.13)	<.0001	1.07 (1.03–1.13)	.001
Potential mediator^b^							
Hemoglobin level (per 1-g/dL increase)	355	1.02 (1.00–1.03)	.07	…		1.02 (1.00–1.04)	.07
Weight (per 10-kg increase)	363	1.05 (1.01–1.09)	.02	…		1.01 (.97–1.05)	.58
Cholesterol level (per 1-mmol/L increase)	357	1.04 (1.00–1.08)	.09	…		0.99 (.95–1.03)	.62
Recent acute coinfection	364	1.11 (.99–1.25)	.08	…		1.09 (.98–1.22)	.11

^a^The log_10_ cfPWV was the outcome in linear regression models, providing model coefficients that correspond to fold (relative) changes when back transformed. This table reflects the inclusion of all relevant variables with a *P* value of < .2 for the association with cfPWV in univariable analyses. There was no association between cfPWV and CD4^+^ T-cell activation (*P*** = **.77), CD8^+^ T-cell activation (*P*** = **.37), CD4^+^ T-cell senescence (*P*** = **.98), and CD8^+^ T-cell senescence (*P*** = **.15).

^b^See Supplementary Figure 2 for the directed acyclic graph and identification of confounders vs mediators.

Abbreviations: BP, blood pressure; CI, confidence interval; HIV, human immunodeficiency virus.

After adjustment for these confounders (Table 2A), there was weak evidence that HIV-infected participants had a 7% greater cfPWV (95% CI, −1%–16%; *P* = .08). After adjustment for confounders and mediators, HIV was significantly associated with cfPWV, with a 12% greater cfPWV (95% CI, 2%–23%) in HIV-infected participants (*P* = .02; Table 2A). The effect of sex weakened with the addition of potential mediators (ie, hemoglobin level, weight, cholesterol level, and recent infection) to the model, but effects of age and diastolic blood pressure remained. cfPWV increased by 2% with every 1-g/dL increase in hemoglobin level (*P* = .07), which may be a marker of plasma viscosity. Concurrent infection at HIV diagnosis was not associated with cfPWV (adjusted fold change, 9%; 95% CI, −2%–21%; *P* = .13). When immune variables were considered in addition to this model (Table 2B), exhausted CD4^+^ and CD8^+^ T cells were each independently associated with cfPWV (*P* = .02), and the independent effect of HIV was lost. HIV remained significantly associated with cfPWV, excluding those with WHO stage 3 and 4 (fold change, 12%; 95% CI, 3%–24%; *P* = .01).

**Table 2B.** Effect of the Addition of T-Cell Exhaustion Markers on the Relationship Between Human Immunodeficiency Virus (HIV) and Carotid Femoral Pulse Wave Velocity (cfPWV)

Predictor	Traditional Risk Factors Model					
	With HIV Status (n = 335)		With CD4^+^ T-Cell Exhaustion (n = 181)		With CD8^+^ T-Cell Exhaustion (n = 270)	
	cfPWV,^a^ Fold Change (95% CI)	*P*	cfPWV,^a^ Fold Change (95% CI)	*P*	cfPWV,^a^ Fold Change (95% CI)	*P*
Age (per 10-y increase)	1.18 (1.13–1.23)	<.0001	1.15 (1.10–1.21)	<.0001	1.15 (1.10–1.20)	<.0001
Female sex (vs male sex)	0.92 (.85–.99)	.02	0.83 (.74–.92)	<.0001	0.88 (.81–.96)	.006
Diastolic BP (per 10–mm Hg increase)	1.07 (1.03–1.13)	.001	1.10 (1.04–1.17)	.01	1.10 (1.05–1.15)	<.0001
Hemoglobin level (per 1-g/dL increase)	1.02 (1.00–1.04)	.07	1.01 (.99–1.04)	.36	1.01 (.99–1.03)	.21
HIV infection	1.12 (1.02–1.23)	.02	1.00 (.86–1.15)	.96	1.05 (.94–1.17)	.40
CD4^+^ T-cell exhaustion % (per 10-pp increase)	…		1.03 (1.00–1.05)	.02	…	
CD8^+^ T-cell exhaustion % (per 10-pp increase)	…		…		1.03 (1.00–1.05)	.02

Abbreviations: BP, blood pressure; CI, confidence interval; HIV, human immunodeficiency virus; pp, percentage point.

^a^The log_10_ cfPWV was the outcome in linear regression models, providing model coefficients that correspond to fold (relative) changes when back transformed. This table reflects the use of backward elimination to select a final model from confounders and mediators in Table 2A and then considers additional effects of CD4^+^ or CD8^+^ T-cell exhaustion (there were similar effects in a model including both; n** = **178). There was no association between cfPWV and CD4^+^ T-cell activation (*P*** = **.77), CD8^+^ T-cell activation (*P*** = **.37), CD4^+^ T-cell senescence (*P*** = **.98), and CD8^+^ T-cell senescence (*P*** = **.15).

### HIV-Related Arterial Stiffness Improves During ART

At week 44, 228 participants with (82%) and 103 (94%) without HIV infection remained in the study. All HIV-uninfected participants were retested for HIV at the week 44 visit, and none had acquired a new infection.

**Figure 2.**
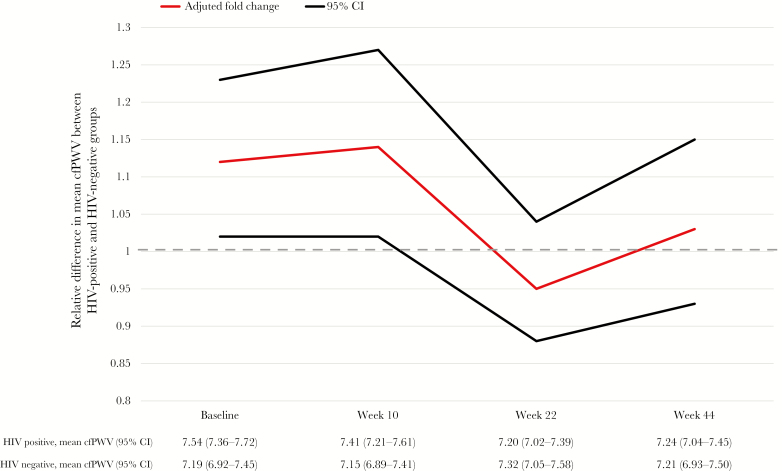
Effect of human immunodeficiency virus (HIV) infection on the carotid femoral pulse wave velocity (cfPWV) over 44 weeks after adjustment for age, sex, diastolic blood pressure, and hemoglobin level. The same model is calculated for each individual time point. CI, confidence interval.

HIV-infected participants still had a significantly higher cfPWV at week 10 (adjusted *P* = .02) but not at week 22 (*P* = .46) or week 44 (*P* = .59; Figure 2). Overall, from enrollment through 44 weeks, cfPWV declined by 9% (95% CI, 4%–15%; *P* = .002) in HIV-infected participants but did not change significantly in HIV-uninfected participants (change, 2%; 95% CI, −7%–12%; *P* = .69; heterogeneity *P* = .04). In a sensitivity analysis, a similar effect of HIV on cfPWV was found at each time point when only including participants who completed the study: fold change at enrollment, 10% (95% CI, 0%–21%); fold change at week 10, 13% (95% CI, 2%–27%; *P* = .022); fold change at week 24, −4% (95% CI, −13%–6%; *P* = .45); and fold change at week 44, 3% (95% CI, −7%–14%; *P* = .60).

For HIV-infected participants, the median CD4^+^ T-cell count increased to 144 cells/μL (IQR, 99–218 cells/μL) at week 44. The mean percentage of CD8^+^ T cells decreased significantly (from 82% [95% CI, 84%–96%] to 60% [95% CI, 54%–67%]; *P* < .0001). The only significant decreases by week 44 involved the mean percentage of activated CD4^+^ T cells (from 74% [95% CI, 62%–86%] to 61% [95% CI, 50%–69%]; *P* < .0001) and the mean percentages of exhausted CD4^+^ T cells (from 54% [95% CI, 31%–67%) to 32% [95% CI, 22%–48%]; *P* < .0001) and exhausted CD8^+^ T cells (from 38% [95% CI, 29%–50%] to 32% [95% CI, 21%–47%]; *P* = .007; Supplementary Table 2).

### Resolution of Higher CD8^+^ T-Cell PD-1 Expression Is Associated With Lower Arterial Stiffness 44 Weeks After ART Initiation

Next, we examined the impact of baseline factors on cfPWV 44 weeks after ART initiation, adjusting for baseline cfPWV (equivalent to predictors for the change in cfPWV), age, sex, baseline hemoglobin level, and diastolic blood pressure (Table 3). There was a trend toward an independent association between a higher percentage of PD-1^+^CD8^+^ T cells at baseline and a lower arterial stiffness 44 weeks after ART initiation (4% decrease in the cfPWV at week 44 for every 10% increase in the percentage of PD-1^+^CD8^+^ T cells at enrollment; *P* = .054). When REALITY intervention arms were added one by one to the same model (adjusted for age, sex, baseline hemoglobin level, diastolic blood pressure, PD-1^+^CD8^+^ T-cell percentage, and baseline cfPWV), there was no evidence of an association between the week 44 cfPWV and random assignment to enhanced infection prophylaxis (fold change vs standard-prophylaxis, 2%; 95% CI, −9%–11%; *P* = .76) or to enhanced nutritional support (fold change vs standard support, 8%; 95% CI, −3%–20%; *P* = .15). However, there was a trend toward a lower adjusted cfPWV at week 44 among the 82 participants (29%) randomly assigned to adjunctive raltegravir for 12 weeks at ART initiation (fold change vs standard ART alone, −11%; 95% CI, −21% to −1%; *P* = .04). This effect of raltegravir on the cfPWV at week 44 was not lost by adding the HIV load at baseline (fold change, −12%; 95% CI, −22%–0%; *P* = .05), week 12 (fold change, −14%; 95% CI, −25%–0%; *P* = .04), or week 24 (fold change, −12%; 95% CI, −24%–1%; *P* = .08).

**Table 3.** Factors Associated With Arterial Stiffness 44 Weeks After Antiretroviral Therapy Initiation Among Human Immunodeficiency Virus–Infected Adults

Factor	Baseline Factors Only (n = 237)		Baseline Factors and Change From Baseline to Week 44^a^ (n = 174)	
	Fold Change (95% CI)	*P*	Fold Change (95% CI)	*P*
Baseline cfPWV (per 1-m/second increase)	2.70 (1.83–4.00)	<.0001	1.15 (1.09–1.22)	<.0001
Age (per 10-y increase)	1.20 (1.11–1.30)	<.0001	1.21 (1.11–1.32)	<.0001
Baseline hemoglobin level (per 1-g/dL increase)	1.03 (1.00–1.06)	.037	1.04 (1.00–1.07)	.027
Baseline PD-1^+^CD8^+^ T-cell % (per 10-pp increase)	0.96 (.91–1.00)	.054	0.99 (.95–1.03)	.71
Change in PD-1^+^CD8^+^ T-cell % over 44 wk (per 10-pp increase)	…		1.02 (1.00–1.05)	.079
Change in CD8^+^ T-cell % over 44 wk (per 10-pp increase)	…		1.04 (.99–1.09)	.13

Abbreviations: CI, confidence interval; cfPWV, carotid femoral pulse wave velocity; pp, percentage point.

^**a**^Including factors with a univariable *P* value of < .05 from Table 4.

Examining the relationship between the change in immune markers and the change in the cfPWV from baseline to week 44, we found that greater decreases in the percentage of CD8^+^ T cells, the percentage of PD-1^+^CD8^+^ T cells, and the proportion of intermediate monocytes were associated with greater improvements in arterial stiffness univariably (*P* = .01, *P* = .03, and *P* = .054, respectively; Table 4). Adjusting for baseline factors and changes in the percentage of CD8^+^ T cells and the percentage of PD-1^+^CD8^+^ T cells, we found that the baseline effect of the percentage of PD-1^+^CD8^+^ T cells on the cfPWV at week 44 was attenuated and that there was instead a trend toward a lower cfPWV at week 44 among participants with a greater decrease in the percentage of PD-1^+^CD8^+^ T cells (*P* = .079; Table 3). Overall, these data suggest that resolution of an initially high proportion of PD-1^+^CD8^+^ T cells is associated with an improvement in arterial stiffness over 44 weeks of ART.

**Table 4.** Change in Clinical and Immune Parameters and Association With Change in Arterial Stiffness 44 Weeks After Enrollment Among Human Immunodeficiency Virus (HIV)–Infected Adults

Parameter	Rho	*P*
Blood pressure (mm Hg)		
Systolic	0.03	.72
Diastolic	0.07	.29
Weight (kg)	−0.09	.21
Creatinine level (µmol/L)	0.01	.88
Hemoglobin level (g/dL)	0.03	.72
HIV load (copies/µL)	−0.08	.28
CD4^+^ T-cell count (cells/µL)	−0.04	.64
CD8^+^ T-cell %	0.21	.01
CD4^+^ to CD8^+^ T-cell ratio	−0.13	.12
CD4^+^ T-cell parameter (% of cells)		
Activated	0.03	.79
Exhausted	0.14	.22
Senescent	0.13	.24
CD8^+^ T-cell parameter (% of cells)		
Activated	−0.03	.72
Exhausted	0.19	.03
Senescent	0.04	.67
Monocyte phenotype (% of cells)		
Classical	−0.16	.07
Intermediate	0.18	.054
Nonclassical	0.01	.91

## DISCUSSION

We have demonstrated that arterial stiffness is increased in Malawian adults with advanced HIV disease during the first 3 months of ART and that T cells expressing PD-1 are associated with this effect. Further, we have shown that this effect is reversible, in a cohort initiating ART with severe immunosuppression and with only modest increases in CD4^+^ T-cell counts during ART. Those with the highest proportion of PD-1^+^CD8^+^ T cells seemed to benefit the most from ART, demonstrating lower arterial stiffness 44 weeks after ART initiation. This is superimposed on a high background prevalence of hypertension.

Hypertension was the most important traditional risk factor for cardiovascular disease [29]. Our findings are consistent with results of studies from the region, which reported a prevalence of 30%–50% in the general population, with as few as 7% aware of their diagnosis [30]. In regions further along the epidemiological transition [11], lifestyle-related CVD risk factors such as obesity and diabetes are becoming more important and are compounded by HIV infection [31]. Intervention to prevent CVD in low-income countries is urgently needed before traditional risk factors intersect with the increased risk associated with HIV.

HIV-infected participants had a 12% higher adjusted arterial stiffness at ART initiation, compared with our HIV-uninfected population. Persistent higher arterial stiffness in the first 3 months of ART is consistent with findings by Benjamin et al, who reported vasculitis as the pathological phenotype of vascular injury during this period, potentially reflecting an immune reconstitution inflammatory syndrome (IRIS)–type phenomenon [32]. Excess mortality during the first 3 months of ART is well recognized in patients initiating ART with very low CD4^+^ T-cell counts [10, 33]. The REALITY trial assessed interventions to reduce this, but even with enhanced infection prophylaxis, 24-week mortality was still 8.9% [34]. Vascular inflammation, driven by high immune activation, may contribute to some of the adverse events seen during the first 3 months of ART in those initiating ART with low CD4^+^ T-cell counts.

In particular, PD-1^+^CD8^+^ T cells have previously been associated with endothelial dysfunction in patients with HIV infection [35–38]. In a cross-sectional study of 358 participants from the SCOPE cohort (of whom 75% had achieved virological suppression during ART), PD-1^+^ T cells were raised during untreated and treated HIV infection, and PD-1^+^CD8^+^ expression was particularly associated with markers of HIV antigenemia, including CD8^+^ T-cell activation and the HIV load [39]. Given the association with reductions in arterial stiffness demonstrated here, the PD-1^+^CD8^+^ expression pathway warrants further investigation. However, in an environment where concurrent acute and latent infections are common, it may be that improvements in PD-1^+^CD8^+^ expression and arterial stiffness were not due to ART and control of HIV alone [40, 41]. Rather, there may have been a protective effect from coadministration of trimethoprim-sulfamethoxazole, preventing coinfections such as those due to malaria parasite or bacteria. A so-called ART care effect may have contributed to improvements over the study period, whereby patients who are engaged in care in a low-income setting experience benefits such as frequent monitoring or better access to care.

Study strengths include prospective follow-up with robust assessment of clinical and cardiovascular measures, as well as a comprehensive longitudinal characterization of both monocyte and T-cell surface activation markers from fresh PBMCs in a large cohort of HIV-infected and uninfected participants. As HIV infection is a generalized epidemic in Malawi, the HIV-infected population is likely to have a traditional cardiovascular risk profile broadly similar to that of HIV-uninfected adults enrolled from the same facility. Further, all but 1 participant received the same standard first-line ART regimen, meaning that choices relating to the specific ART regimen cannot be confounders of the associations identified.

Limitations include the fact that cfPWV has not been validated to predict cardiovascular events in low-income areas of SSA; however, cfPWV has been validated robustly elsewhere [42, 43]. The relatively small differences identified in continuous cfPWV may have uncertain clinical relevance, and our hypothesized poor outcome (cfPWV >12 m/second) was rare, likely because our power calculations were based on studies from high-income settings and on much older populations than the HIV-infected population in SSA [44–46]. Overall, our study is still the largest to address this issue in the region, and it demonstrates that HIV has an effect on the risk of cardiovascular events that is similar in magnitude to that of traditional risk factors, such as age (18% per 10-year increase) and blood pressure (9% per 10–mm Hg increase).

Our HIV-uninfected population was selected to reflect generally healthy adults with similar sociodemographic characteristics. Comparisons with our severely immunosuppressed HIV-infected population therefore reflect the extremes, and this also limits the generalizability of our study to unselected HIV populations. However, WHO disease stage, CD4^+^ T-cell count, and presence of coinfections and their clinical markers were not independently associated with arterial stiffness, suggesting that the effects of HIV may exist across the disease spectrum. Acute coinfections at the time of diagnosis were rare, limiting our power to assess their effects, but they may have contributed to an IRIS-type phenomenon. To avoid overburdening potentially clinically unwell patients who urgently needed to start ART, enrollment assessments were performed 2 weeks after ART initiation. As early mortality is high and some limited normalization may have occurred, our findings are likely a best-case scenario [24].

Last, the size of the effect of PD-1^+^CD8^+^ T cells on arterial stiffness was modest and is likely to represent one component of several concurrent complex mechanisms involved in the pathogenesis of endothelial damage in people living with HIV in this setting. Nevertheless, exhaustion was the strongest immune predictor of cfPWV on ART, and other immunophenotyping parameters did not have independent effects after adjusting for PD-1 expression.

This is the first time that the dynamics of arterial stiffness in the first few months of ART have been documented longitudinally and related to cellular markers of immune activation in HIV-infected adults. We have confirmed hypertension as the primary CVD risk factor in the region and demonstrated that consequences of immune activation can be reversed irrespective of CD4^+^ T-cell count recovery, with early benefits for vasculature. Further studies into infection-driven immune activation, as a risk factor for CVD, are warranted, but guidelines for prevention of CVD in HIV-infected individuals are urgently needed and should focus on hypertension reduction and close monitoring for those starting ART at lower CD4^+^ T-cell counts.

## Supplementary Data

Supplementary materials are available at *The Journal of Infectious Diseases* online. Consisting of data provided by the authors to benefit the reader, the posted materials are not copyedited and are the sole responsibility of the authors, so questions or comments should be addressed to the corresponding author.

jiz015_suppl_Supplementary_Material

## Notes

***Acknowledgments.* **We thank the participating patients and their families; the staff in the ART clinic and Department of Medicine at Queen Elizabeth Central Hospital, Blantyre, Malawi; and the REALITY trial group (Supplementary Materials) for their support with study design and implementation.

***Financial support.* **This work was supported by the Wellcome Trust (grant number 099934/Z/12/A to C. K., strategic award to the MLW Clinical Research Programme, and core support [via MC_UU_12023/23 and MC_UU_12023/26] to the MRC Clinical Trials Unit at UCL). Cipla, Gilead Sciences, ViiV Healthcare/GlaxoSmithKline, and Merck Sharp and Dohme donated drugs for use in the REALITY trial.

Presented in part: CROI, Boston, Massachusetts, March 2017. Abstract 952.

***Potential conflicts of interest.* **All authors: No reported conflicts of interest. All authors have submitted the ICMJE Form for Disclosure of Potential Conflicts of Interest. Conflicts that the editors consider relevant to the content of the manuscript have been disclosed.
