# Supplementary material for: HIV-Related Arterial Stiffness in Malawian Adults Is Associated With the Proportion of PD-1–Expressing CD8+ T Cells and Reverses With Antiretroviral Therapy
Source: J Infect Dis. 2019 Jan 9;219(12):1948–58. doi: 10.1093/infdis/jiz015 (PMC6534190; doi:10.1093/infdis/jiz015)
Supplement: jiz015_suppl_Supplementary_Material [file jiz015_suppl_supplementary_material.pdf]

Supplementary Material

Supplementary Figure 1A. T cell gating strategy

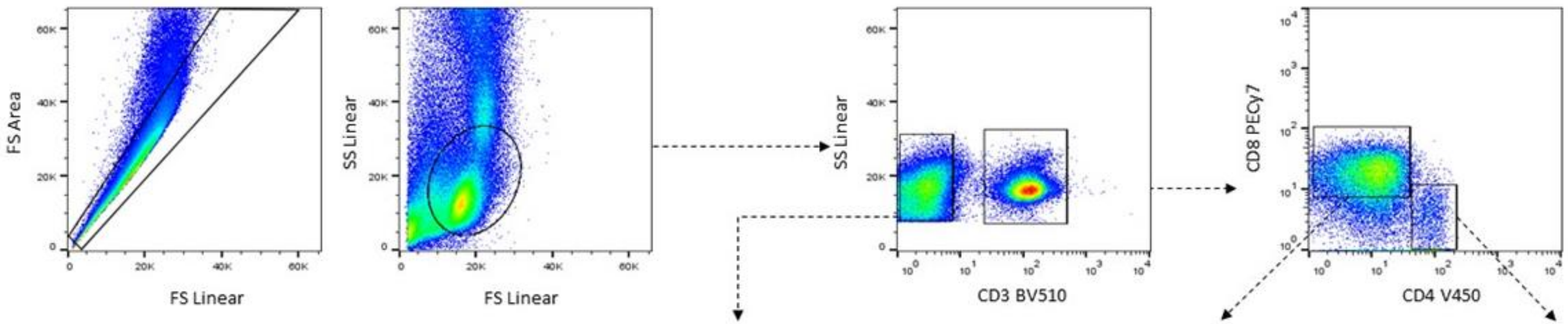

All PBMCs were gated to identify singlets. Lymphocytes were then selected based on light properties. T cells were identified as CD3 positive and were then separated into CD4 positive and CD8 positive. The CD3 negative population was used to identify clear negative populations for HLA-DR, CD38, PD-1 and CD57 which corresponded well with FMOs

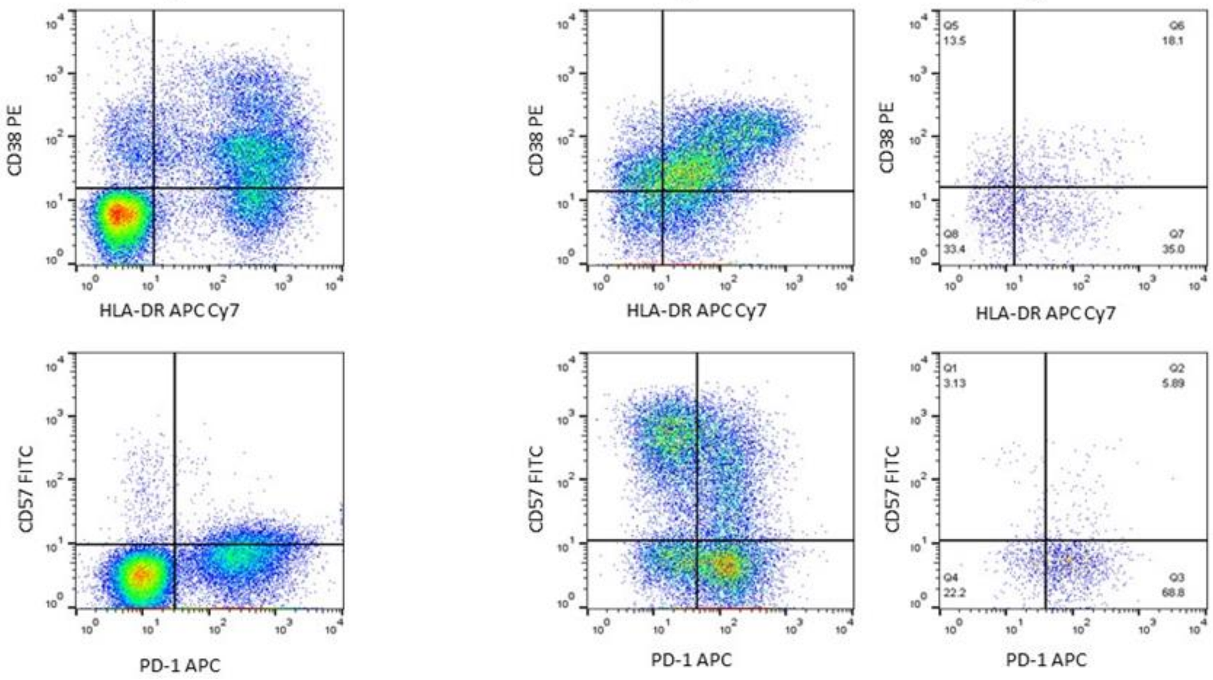

Supplementary Figure 1B. Monocyte gating strategy

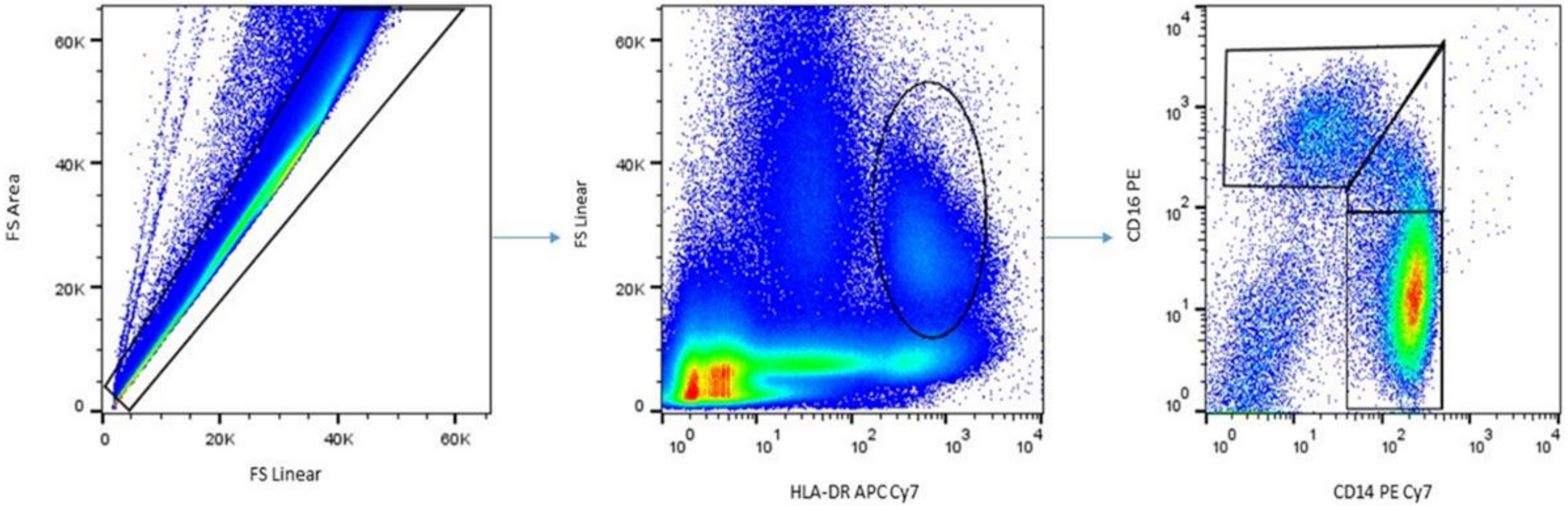

All PBMCs were gated to identify singlets. Monocytes were identified based on HLA-DR expression and size. Monocyte subsets were gated according to previously published guidance [Passlick et al. **Blood**. 1989]

Supplementary Material Figure 2. Direct Acyclic Graph for mediators of cfPWV

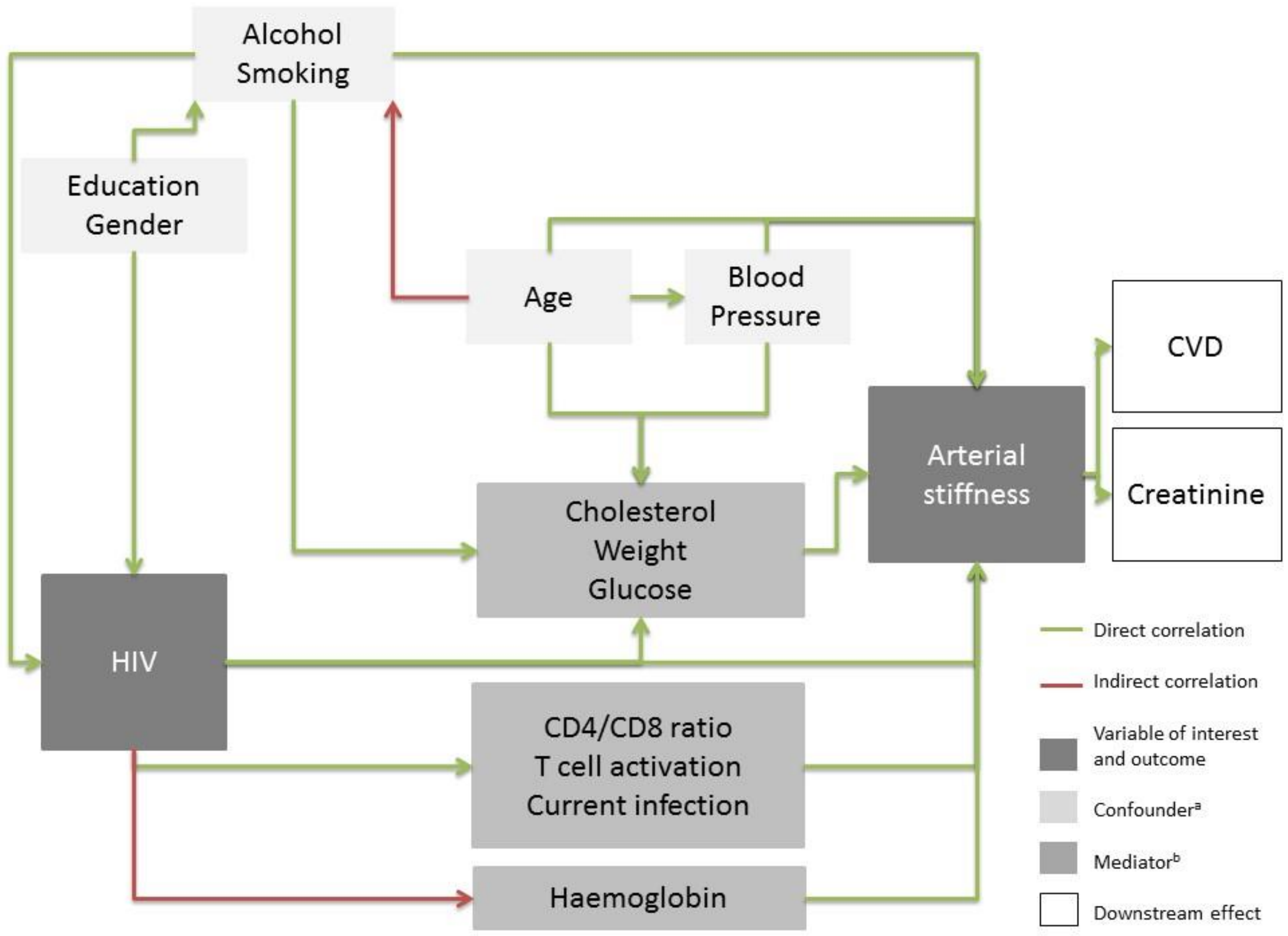

[a]A confounder was judged to be any variable which was associated with both HIV and arterial stiffness but was not judged *a priori* to lie on the mechanistic pathway between them. [b]A mediator was a variable that was judged *a priori* to lie on the mechanistic pathway between HIV and arterial stiffness.

Supplementary Material Figure 3. Absolute cfPWV values by age and HIV status

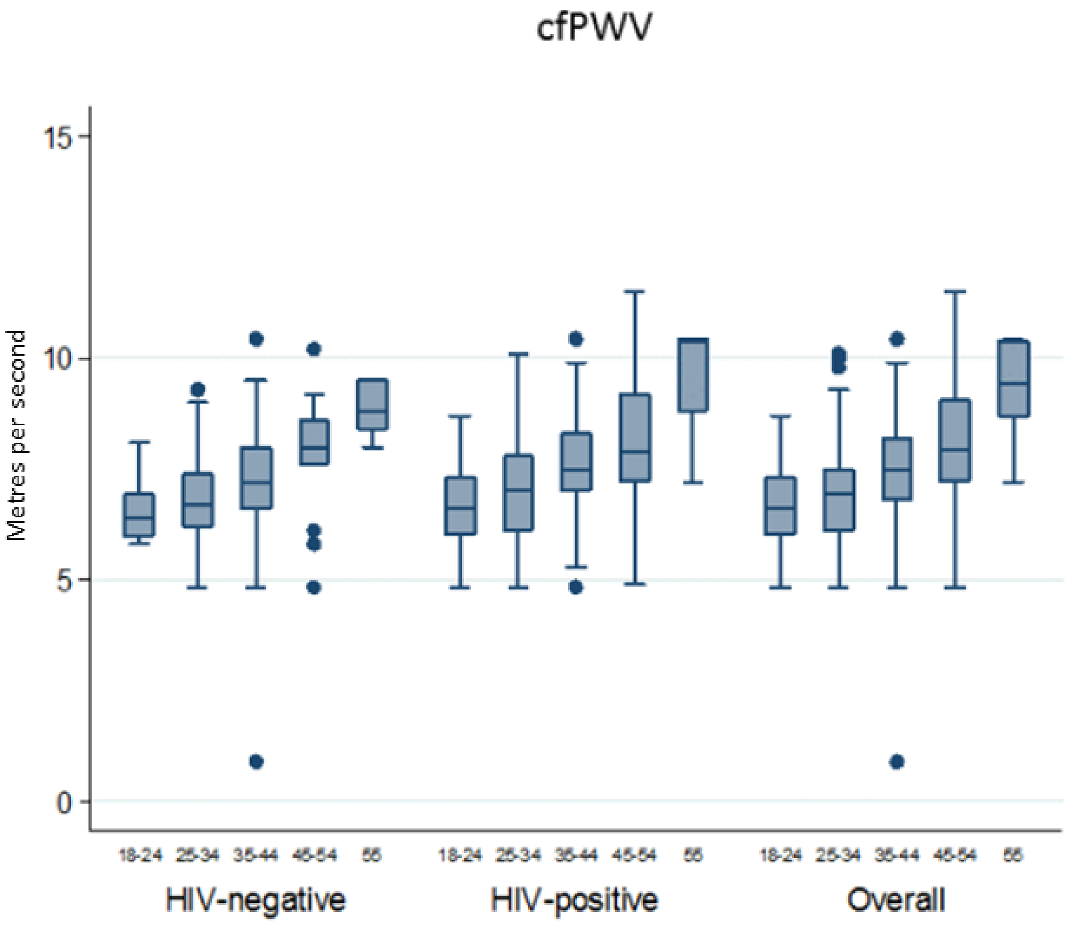

Supplementary Material Table 1. Reasons screened but not recruited for HIV infected adults

Reason not recruited	Number screened but not recruited
CD4 >100	1477
Declined	117
Died before enrolment visit	42
Received ART from National Program before enrolment	39
Not a Blantyre resident	23
Laboratory Machine failure	16
Lost	14
Recruited to another study	12
Too unwell	9
Pregnant	8
Received ART from private system	2
Unknown	69
Total	1828

Supplementary Material Table 2. Immune activation markers at enrolment and week 44 by HIV status

		Enrolment			Week 44	
		**HIV uninfected n=94**	**HIV infected n=202**	**p value[a]**	**HIV Infected n=190**	**p value[b]**
**Absolute CD4 count (cells/µL)**		NA	42 (18 – 62)	NA	144 (99 – 219)	NA
**HIV viral load (copies/ml)**		NA	113,560 (41,747 – 293,663)	NA	NA	NA
**CD4 T cells**	**CD4/CD8 ratio**	1.39 (1.02 – 2.15)	0.09 (0.04 – 0.20)	<0.00001	0.14 (0.06 – 0.23)	0.007
	**%CD4**	58.1 (50.5 -68.3)	8.2 (4.1 – 16.5)	<0.0001	8.11 (4.1 – 12.4)	0.89
	**CD4 Activated**	53.5 (40.5 - 65.0)	74.5 (61.5 – 85.7)	<0.0001	60.7 (49.7 – 69.3)	<0.0001
	**CD4 Exhausted**	15.0 (9.3 – 23.6)	54.0 (31.3 – 66.9)	<0.0001	31.9 (21.9 – 48.1)	<0.0001
	**CD4 Senescent**	6.7 (3.6 – 9.03)	14.8 (9.0- 23.9)	<0.0001	14.7 (9.0 – 23.3)	0.99
**CD8 T cells**	**%CD8**	41.1 (31.7 – 50.0)	81.8 (83.5 – 95. 9)	<0.0001	60.4 (54.3 – 67.0)	<0.0001
	**CD8 Activated**	59.6 (39.8 – 72.7)	78.6 (64.5 – 87.6)	<0.0001	75.3 (66.7 – 83.8)	0.21
	**CD8 Exhausted**	20.9 (12.2 – 29.4)	37.7 (28.6 – 49.5)	<0.0001	31.7 (20.6 – 47.4)	0.007
	**CD8 Senescent**	39.7 (26.5 – 53.4)	53.6 (43.9 – 64.1)	<0.0001	61.0 (47.3 – 68.3)	0.001
**Monocytes**	**Classical**	75.4 (64.9 – 80.7)	75.5 (66.3 – 82.5)	0.79	74.8 (67.1 – 80.7)	0.90
	**Intermediate**	9.4 (6.7 – 14.4)	9.9 (6.0 – 13.1)	0.99	8.5 (6.0 – 12.7)	0.13
	**Non-classical**	13.1 (9.8 – 22.0)	13.6 (9.0 – 20.6)	0.59	15.3 (10.9 – 21.3)	0.07

[a]Comparing HIV infected versus HIV uninfected participants at enrolment (2 weeks post ART initiation), Wilcoxon Ranksum. [b]Comparing HIV infected participants at enrolment to HIV infected participants at week 44, Paired Wilcoxon.

The REALITY trial group consists of:

Participating Centres: Joint Clinical Research Centre (JCRC), Kampala, Uganda (coordinating centre for Uganda): P Mugyenyi, C Kityo, V Musiime, P Wavamunno, E Nambi, P Ocitti, M Ndigendawani. JCRC, Fort Portal, Uganda: S Kabahenda, M Kemigisa, J Acen, D Olebo, G Mpamize, A Amone, D Okweny, A Mbonye, F Nambaziira, A Rweyora, M Kangah and V Kabaswahili. JCRC, Gulu, Uganda: J Abach, G Abongomera, J Omongin, I Aciro, A Philliam, B Arach, E Ocung, G Amone, P Miles, C Adong, C Tumsuiime, P Kidega, B Otto, F Apio. JCRC, Mbale, Uganda: K Baleeta, A Mukuye, M Abwola, F Ssennono, D Baliruno, S Tuhirwe, R Namisi, F Kigongo, D Kikyonkyo, F Mushahara, D Okweny, J Tusiime, A Musiime, A Nankya, D Atwongyeire, S Sirikye, S Mula, N Noowe. JCRC, Mbarara, Uganda: A Lugemwa, M Kasozi, S Mwebe, L Atwine, T Senkindu, T Natuhurira, C Katemba, E Ninsiima, M Acaku J Kyomuhangi, R Ankunda, D Tukwasibwe, L Ayesiga. University of Zimbabwe Clinical Research Centre, Harare, Zimbabwe: J Hakim, K Nathoo, M Bwakura-Dangarembizi, A Reid, E Chidziva, T Mhute, GC Tinago, J Bhiri, S Mudzingwa, M Phiri, J Steamer, R Nhema, C Warambwa, G Musoro, S Mutsai, B Nemasango, C Moyo, S Chitongo, K Rashirai, S Vhembo, B Mlambo, S Nkomani, B Ndemera, M Willard, C Berejena, Y Musodza, P Matiza, B Mudenge, V Guti. KEMRI Wellcome Trust Research Programme, Kilifi, Kenya: A Etyang, C Agutu, J Berkley, K Maitland, P Njuguna, S Mwaringa, T Etyang, K Awuondo, S Wale, J Shangala, J Kithunga, S Mwarumba, S Said Maitha, R Mutai, M Lozi Lewa, G Mwambingu, A Mwanzu, C Kalama, H Latham, J Shikuku, A Fondo, A Njogu, C Khadenge, B Mwakisha. Moi University Clinical Research Centre, Eldoret, Kenya: A Siika, K Wools-Kaloustian, W Nyandiko, P Cheruiyot, A Sudoi, S Wachira, B Meli, M Karoney, A Nzioka, M Tanui, M Mokaya, W Ekiru, C Mboya, D Mwimali, C Mengich, J Choge, W Injera, K Njenga, S Cherutich, M Anyango Orido, G Omondi Lwande, P Rutto, A Mudogo, I Kutto, A Shali, L Jaika, H Jerotich, M Pierre. Department of Medicine and Malawi-Liverpool Wellcome Trust Clinical Research Programme, College of Medicine, Blantyre, Malawi: J Mallewa, S Kaunda, J Van Oosterhout, B O'Hare, R Heyderman, C Gonzalez, N Dzabala, C Kelly, B Denis, G Selemani, L Nyondo Mipando, E Chirwa, P Banda, L Mvula, H Msuku,

M Ziwoya, Y Manda, S Nicholas, C Masesa, T Mwalukomo, L Makhaza, I Sheha, J Bwanali, M Limbuni.

Trial Coordination and Oversight: MRC Clinical Trials Unit at UCL, London, UK: D Gibb, M Thomason, AS Walker, S Pett, A Szubert, A Griffiths, H Wilkes, C Rajapakse, M Spyer, A Prendergast, N Klein. Data Management Systems: M Rauchenberger, N Van Looy, E Little, K Fairbrother.

Social Science Group: F Cowan, J Seeley, S Bernays, R Kawuma, Z Mupambireyi.

Independent REALITY Trial Monitors: F Kyomuhendo, S Nakalanzi, J Peshu, S Ndaa, J Chabuka, N Mkandawire, L Matandika, C Kapuya.

Trial Steering Committee: I Weller (Chair), E Malianga, C Mwansambo, F Miiro, P Elyanu, E Bukusi, E Katabira, O Mugurungi, D Gibb, J Hakim, A Etyang, P Mugyenyi, J Mallewa.

Data Monitoring Committee: T Peto (Chair), P Musoke, J Matenga, S Phiri.

Endpoint Review Committee (independent members): H Lyall (Co-Chair), V Johnston (Co-Chair), F Fitzgerald, F Post, F Ssali, A Prendergast, A Arenas-Pinto, A Turkova, A Bamford.
